# *UDock2*: interactive real-time multi-body protein–protein docking software

**DOI:** 10.1093/bioinformatics/btad609

**Published:** 2023-10-04

**Authors:** Cyprien Plateau-Holleville, Simon Guionnière, Benjamin Boyer, Brian Jiménez-Garcia, Guillaume Levieux, Stéphane Mérillou, Maxime Maria, Matthieu Montes

**Affiliations:** XLIM, UMR CNRS 7252, University of Limoges, 87000 Limoges, France; Laboratoire GBCM, EA 7528, Conservatoire National des Arts et Métiers, Hésam Université, 75003 Paris, France; Laboratoire GBCM, EA 7528, Conservatoire National des Arts et Métiers, Hésam Université, 75003 Paris, France; Zymvol Biomodeling, 08010 Barcelona, Spain; CEDRIC, EA 4626, Conservatoire National des Arts et Métiers, Hésam Université, 75003 Paris, France; XLIM, UMR CNRS 7252, University of Limoges, 87000 Limoges, France; XLIM, UMR CNRS 7252, University of Limoges, 87000 Limoges, France; Laboratoire GBCM, EA 7528, Conservatoire National des Arts et Métiers, Hésam Université, 75003 Paris, France; Institut Universitaire de France (IUF), Paris 75005, France

## Abstract

**Motivation:**

Protein–protein docking aims at predicting the geometry of protein interactions to gain insights into the mechanisms underlying these processes and develop new strategies for drug discovery. Interactive and user-oriented manipulation tools can support this task complementary to automated software.

**Results:**

This article presents an interactive multi-body protein–protein docking software, *UDock2*, designed for research but also usable for teaching and popularization of science purposes due to its high usability. In *UDock2*, the users tackle the conformational space of protein interfaces using an intuitive real-time docking procedure with on-the-fly scoring. *UDock2* integrates traditional computer graphics methods to facilitate the visualization and to provide better insight into protein surfaces, interfaces, and properties.

**Availability and implementation:**

*UDock2* is open-source, cross-platform (Windows and Linux), and available at http://udock.fr. The code can be accessed at https://gitlab.com/Udock/Udock2.

## 1 Introduction

The critical involvement of protein–protein interactions in essential biological processes, including immunity and inflammation, has been well-established (Braun and Gingras 2012). To explore the geometry of these interactions, researchers have utilized protein–protein docking simulations, which aim to predict the relative position of the involved proteins. Such simulations have been shown to be effective in ranking the predicted geometries based on their binding energy, calculated using a molecular mechanics force-field (Meng *et al.* 2011). By providing insight into the spatial arrangement of proteins and their binding sites, protein–protein docking simulations offer a valuable tool for understanding the mechanisms underlying complex biological processes.

The manipulation and visualization of molecular bodies through an ergonomic and intuitive user interface in docking software still represent a challenge. Different interactive docking methods have been released over time that notably suffers from limited usability and/or dependency on proprietary or expensive hardware (Daunay *et al.* 2007, Zonta *et al.* 2008, Férey *et al.* 2009, Matthews *et al.* 2019). Even though several works presented alternative approaches (Lu *et al.* 2005, Levieux *et al.* 2014, Deeks *et al.* 2020, Iakovou *et al.* 2020) for the popularization of such methods, combining performance, user-oriented features, and comprehensive feedback for interactive manipulation is still a complicated task.

In this article, we describe *UDock2*, the new version of *UDock* (Levieux *et al.* 2014), an intuitive and interactive multi-body protein–protein docking method oriented toward its ease of usability.

In order to broaden its potential audience, *UDock2* is designed to be used on low-end computers. It therefore relies on standard CPU computing strategies and do not use any feature set restricted to modern GPUs. The main *UDock* features were retained to be integrated into a research workflow with the following extended features:

- To offer up-to-date features, a complete rewrite has been achieved which comes with several user experience improvements thanks to recent computer graphics strategies.
- We provide the ability to study complex molecular systems, especially through the support of multi-body molecular docking thanks to an adapted energy computation scheme.
- We introduce an extended navigation allowing to ease the exploration of molecular complexes by orienting the camera relatively to the molecular surface and user inputs.
- Since *UDock2* is also designed for educational and popularization of science purposes, we improved the gamification of the molecular docking sequence by providing a more playful experience.

## 2 *UDock2*’s workflow

Protein–protein docking simulation is usually computationally intensive and performed by automated software lacking intuitive user interaction features. *UDock2* has been designed to take advantage of the possible solutions proposed interactively by the users, as a part of a protein–protein interactions exploration pipeline.

*UDock2* takes as input all-atom *mol2* format files that include atomic partial charges to provide a way to explore, visualize, understand, and manipulate molecular entities. The manipulation of molecules in the 3D environment is oriented toward binding score awareness to guide the exploration. This helps the understanding of the complex phenomena involved in molecular binding. Proposed geometries can be exported in any molecular file format supported by the Chemfiles library (Fraux *et al.* 2020). Finally, *UDock2*’s exported files can be used in a molecular simulation or molecular visualization software in order to pursue the analysis. Exploratory geometries can afterward be optimized using molecular simulation software to produce accurate solutions. This development has been achieved with plain C++ and OpenGL. The Bullet Physics SDK (http://bulletphysics.org) (Coumans) was used to handle the manipulation of molecular objects and the collision meshes. To support the use of UDock2, we provide a tutorial video as supplementary material.

## 3 Main features

In the following parts, we describe *UDock2*’s main features and their contribution to the general goals of the software.

### 3.1 Molecular data computation

The goal of protein docking is to identify the best geometry of the complex by optimizing the binding energy between the two or more partners involved in the interaction. We then selected the protein’s shape and its electrostatics as the representation provided in *UDock2*.

The global shape of the proteins involved in the interaction is represented using their Solvent-Excluded Surface (SES) (Richards 1977). As *Udock2* is designed to be used on low-end computers, we improve its construction using a time-tested grid-based generation method which has been leveraged several times (Hermosilla *et al.* 2017, Martinez *et al.* 2019). It is based on the Marching Cubes algorithm (Lorensen and Cline 1987) and provides a voxel classification through atom and probe distance. In addition, we introduce an adaptive computation scheme preserving the ratio between performance and smoothness of the resulting mesh. The grid cell size is determined thanks to an empirical equation based on the bounding box’s dimensions $r_{c}=\frac{1}{3}(s_{x}+s_{y}+s_{z})\cdot 10^{-2}$. This simple but effective algorithm provides an approximated coarse SES mesh in a small computation time in line with *Udock2* purpose.

The indication of the electrostatic potential is finally provided by a per-triangle averaging of the atomic partial charges giving a smooth result across the surface.

### 3.2 Scoring multi-body docking

In *UDock2*, the goal is to find the stablest conformation possible between the multiple proteins involved in the docking process. To evaluate the stability of the proposed complex, a function approximating the binding energy with reduced computational cost is used to produce a score, giving a real-time feedback to the user. Therefore, the user aims at minimizing this score.

Various scoring functions have been developed for protein–protein docking (Janin *et al.* 2003, Vakser 2014) based on molecular mechanics force-fields, statistical potentials, or empirically derived contributions. We chose to keep the scoring function used in *Udock*.

Soft repulsive and attractive van der Waals forces are represented using the 8–6 Lennard–Jones potential model:


(1) $$E_{p}\left(i,j\right)=3\epsilon {\left(\frac{r_{i}+r_{j}}{d_{ij}}\right)}^{8}-4\epsilon {\left(\frac{r_{i}+r_{j}}{d_{ij}}\right)}^{6},$$

with *i* and *j* as the involved atoms, *r* as their radius, $d_{ij}$ as their relative distance, and $\epsilon =\sqrt{\epsilon_{i}\epsilon_{j}}$ as Berthelot’s empirical weighting of the interaction between both atoms.

Longer-range electrostatics are defined thanks to Coulomb’s law such as:


(2) $$F\left(i,j\right)=332.0522\frac{c_{i}c_{j}}{E_{0}d_{ij}}.$$

with *c* as an atom charge, 332.0522 as a conversion factor based on AMBER23 documentation (Case *et al.* 2023) and resulting in kcal/mol, and $E_{0}=20$ as a distance-dependent dielectric constant (Meng *et al.* 2011, Levieux *et al.* 2014).

The sum of both terms then gives an estimation of the energy between two atoms *i* and *j*:


(3) $$E\left(i,j\right)=F\left(i,j\right)+E_{p}\left(i,j\right).$$

All atom pairs contribute to the resulting binding score according to their respective distances that are calculated in real-time. However, reaching an interactive display of the binding score during multi-body docking simulation is highly reliant on the execution of the scoring function. This core feature can be settled by using the scoring function on every molecule couple and then summed to provide a score for the complete scene state. If achieved on a per-atom basis, it would lead to linear complexity levels and thus limit its use for large molecular systems. Even if this process could easily be accelerated using the massively parallel architecture of modern GPUs, this would prevent us from reaching our audience target. Thus, we restricted the sum for every atom of the first molecule to their neighborhood in the second with a configurable threshold which value is defaulted to 12 Å as commonly achieved for electrostatic interaction in molecular simulation (Lagardère *et al.* 2018). This value is also selected based on previous study showing that it is optimal for docking simulations scoring (Sinha *et al.* 2012). Finally, we use a grid-based acceleration structure, known for joining implementation simpleness and good results in this kind of application. This computation method allows us to load molecules with tens of thousands of atoms without suffering from performance issues. Users can hence rely on immediate and interactive feedback from their actions.

We leverage the fast scoring computation to provide automatic rigid-body optimization of the complex. To do so, we apply random modifications of the selected molecule’s position and rotation to refine the overall score in an iterative fashion repeated as many times as configured by the user. This process allows the user to improve a starting configuration to further minimize the binding energy.

### 3.3 User interface

A challenging aspect of working with 3D molecular scenes is to provide a clear vision of the global orientation and conformation of the molecules and the ability to inspect the details of interacting molecular surfaces. To address this issue, we provide two first-person camera modes. The first one offers 6 degrees of freedom, while the second is oriented toward close-distance visualization thanks to a local trackball system.

To prevent the binding score to be extreme (i.e. when $d_{ij}$ is too small in equation 3), a collision mesh corresponding to the SES is used in a physics engine. This strategy not only eases the interaction of the user with the complex but also enables the creation of a manipulation tool. The grapnel system based on these colliders and used in *UDock* has then been kept as the main molecule manipulation tool of the software.

Finally, the energy score is accessible to the user at the top of the window. The display is updated in real-time for every change made on the conformation. It gives progress insights and thus guides the manipulation.

### 3.4 Real-time visualization

**Figure 1.**
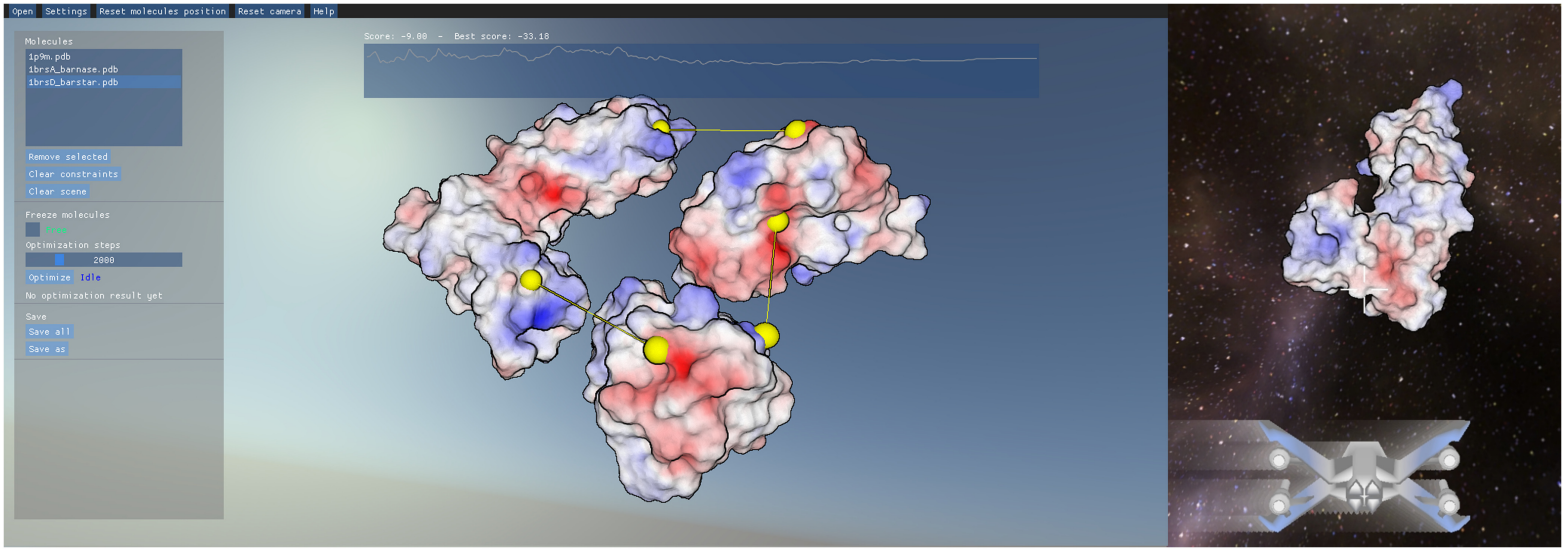
Illustrations of a multi-body docking experience using *UDock2* (left) and the avatar-based navigation (right). On top, the estimated binding score is displayed in real-time guiding the user toward its minimization. On the left part of the window is a panel including different manipulation and object selection options. In yellow, grapnels are used as constraints to guide molecular docking.

Many visualization software provides a fixed flat colored background. To improve user experience, we integrated an environment map as the background of the scene. For instance, the visualization benefits from the information of the skyline offered by a realistic sky (Hosek and Wilkie 2012), as illustrated in Fig. 1, left. It leads to a better perception of the camera orientation which eases the exploration of the scene.

To further enhance the readability of the SES, we added several shading effects. The surface is colored based on the electrostatic potential computed previously. By default, as usual for electrostatic surface potentials, red is used for negative charge values, white for neutral charge values, and blue for positive charge values. This coloration offers useful information to the users to drive the molecular docking process, as they try to match globally positive areas with globally negative areas. Besides the coloring process, the rendering of molecular shapes is stylized through dedicated passes targeting better readability and perception. It is composed of a black contour line from an edge emphasizing filter on the depth texture in addition to a reflection mapping to shade molecules according to the environment map. We, however, kept this last shading operation optional to give a raw diffuse shading if it better suits the user’s needs.

### 3.5 Avatar-based navigation

As illustrated in Fig. 1, right, *UDock2* offers an interactive video-game-derived third-person view that is controllable via a classical mouse and keyboard or a gamepad through a spaceship-like avatar. This allows direct and close exploration of the protein’s surfaces and their interactions. The avatar is integrated with the physics engine permitting its movements to have inertia. This feature is intended for less precise but more comfortable navigation. It also means that the avatar has a physical presence and can collide with molecules. The user can interact with the molecular scene thanks to a variety of actions: throw a spherical collider to move molecules or place grapnel anchors on their surface. These additional features are useful to access younger audiences and can be the basis of a gamification process for molecular docking.

## 4 Conclusion

*UDock2*, is a real-time multi-body protein–protein docking software with optimized usability thanks to traditional computer–human interaction and computer graphics features adapted to the task. These features not only ease both the learning curve of *UDock2* and its day-to-day use but also its ability to support the understanding of molecular interactions. *UDock2* is designed to be integrated as an early step of a molecular modeling pipeline and can provide useful support for the exploration of different hypotheses of complex multi-body protein–protein interactions. It also targets education and the popularization of science.

## Supplementary Material

btad609_Supplementary_Data
